# Prenatal lipopolysaccharide exposure induces anxiety-like behaviour in male mouse offspring and aberrant glial differentiation of embryonic neural stem cells

**DOI:** 10.1186/s11658-023-00480-7

**Published:** 2023-08-17

**Authors:** Chie-Pein Chen, Pei-Chun Chen, Yu-Ling Pan, Yi-Chao Hsu

**Affiliations:** 1 Division of High Risk Pregnancy, Department of Obstetrics and Gynecology, MacKay Memorial Hospital, Taipei, Taiwan; 2 Department of Medical Research, MacKay Memorial Hospital, Taipei, Taiwan; 3 Department of Audiology and Speech-Language Pathology, MacKay Medical College, New Taipei City, Taiwan; 4 Institute of Biomedical Sciences, MacKay Medical College, New Taipei City, Taiwan

**Keywords:** Lipopolysaccharide, Prenatal infection, Embryonic stem cell, Neural stem cell, Oligodendrocyte differentiation, *ApoB*, *ApoE*

## Abstract

**Background:**

Prenatal infection has been implicated in the development of neuropsychiatric disorders in children. We hypothesised that exposure to lipopolysaccharide during prenatal development could induce anxiety-like behaviour and sensorineural hearing loss in offspring, as well as disrupt neural differentiation during embryonic neural development.

**Methods:**

We simulated prenatal infection in FVB mice and mouse embryonic stem cell (ESC) lines, specifically 46C and E14Tg2a, through lipopolysaccharide treatment. Gene expression profiling analyses and behavioural tests were utilized to study the effects of lipopolysaccharide on the offspring and alterations in toll-like receptor (TLR) 2-positive and TLR4-positive cells during neural differentiation in the ESCs.

**Results:**

Exposure to lipopolysaccharide (25 µg/kg) on gestation day 9 resulted in anxiety-like behaviour specifically in male offspring, while no effects were detected in female offspring. We also found significant increases in the expression of GFAP and CNPase, as well as higher numbers of GFAP + astrocytes and O4+ oligodendrocytes in the prefrontal cortex of male offspring. Furthermore, increased scores for genes related to oligodendrocyte and lipid metabolism, particularly *ApoE*, were observed in the prefrontal cortex regions. Upon exposure to lipopolysaccharide during the ESC-to-neural stem cell (NSC) transition, *Tuj1*, *Map2*, *Gfap*, *O4*, and *Oligo2* mRNA levels increased in the differentiated neural cells on day 14. In vitro experiments demonstrated that lipopolysaccharide exposure induced inflammatory responses, as evidenced by increased expression of *IL1b* and *ApoB* mRNA.

**Conclusions:**

Our findings suggest that prenatal infection at different stages of neural differentiation may result in distinct disturbances in neural differentiation during ESC—NSC transitions. Furthermore, early prenatal challenges with lipopolysaccharide selectively induce anxiety-like behaviour in male offspring. This behaviour may be attributed to the abnormal differentiation of astrocytes and oligodendrocytes in the brain, potentially mediated by ApoB/E signalling pathways in response to inflammatory stimuli.

**Supplementary Information:**

The online version contains supplementary material available at 10.1186/s11658-023-00480-7.

## Introduction

Approximately 10% of all live births are preterm [1]. Prenatal infection-induced preterm birth can lead to neonatal mortality and morbidity. Preterm birth is a critical risk factor for adverse neurological effects in preterm infants [2], who may develop a spectrum of serious cognitive and neurobehavioural disorders in adulthood [3, 4], such as autism spectrum disorders (ASD) in CD1 mice [5, 6], anxiety in C57BL/6 mice [7], and sensorineural hearing loss in clinical settings [8]. However, the mechanisms underlying the neurobehavioural effects of such damage in preterm infants remain unclear. Furthermore, it remains to be investigated whether other mouse strains, such as FVB/NCrlBltw, also exhibit similar phenotypes upon prenatal exposure to lipopolysaccharide (LPS). Notably, increased levels of proinflammatory cytokines, such as tumour necrosis factor (TNF) α, interleukin (IL) 1β, IL-6 and interferon (IFN) γ, in amniotic fluid or cord blood of preterm offspring are associated with the development of neurobehavioural abnormalities in adulthood [9, 10]. However, it is unclear whether prenatal infection during early embryonic stages can have damaging effects on embryonic neural development.

Neural stem cells (NSCs) are crucial for neurogenesis and neural development. They can self-renew and differentiate into key cell types in the cental nervous system, such as neurons, astrocytes and oligodendrocytes, both in vitro and in vivo. In various disease models, NSCs respond to central nervous system trauma, such as brain damage, by differentiating and migrating to the site of injury [11–14]. In damaged central nervous systems, NSCs have been demonstrated not only to serve as a source of newly generated cells, but also potentially provide trophic support to other cells [15, 16]. Toll-like receptors (TLRs) play important roles in the innate immune system, recognizing the conserved molecular structures found in various pathogens [17]. TLR2 and TLR4 are expressed in adult NSCs isolated from the rat subventricular zone of brains. Notably, the mRNA levels of TLR2 and TLR4 are upregulated by TNFα and IFNγ, respectively [18]. However, TLR2 and TLR4 have distinct effects on the proliferation and differentiation of NSCs and neural progenitor cells [19, 20]. The roles of TLR2 and TLR4 in prenatal infection during early neural ectoderm development and neurogenesis need to be elucidated. It has been shown that embryonic stem cells (ESCs) can serve as an unlimited source of NSCs and various types of neural cells. Sox1-GFP ES cells (46C) are genetically-modified ES cells with eGFP knock-in in one allele of the *Sox1* gene. During the NSC differentiation stage, eGFP is activated under the expression of *Sox1* [21]. This NSC-specific reporter ES line provides an attractive model for the studying of ES cell neural differentiation [22].

Prenatal LPS exposure increases the brain’s sensitivity to subsequent hypoxic–ischaemic events and brain injury in infancy [23]. TLR4 serves as the main receptor for pathogen-associated molecular patterns. In humans, upon activation by LPS, TLR4 can substantially contribute to proinflammatory immune responses [24]. It has been demonstrated that maternal inflammation induced by LPS treatment in pregnant CD1 mice on embryonic day 9 results in offspring with an ASD-like phenotype, brain overgrowth, hyperproliferation of brain NSCs, and an increased population of forebrain microglia during adulthood [25]. Prenatal infection or exposure to LPS may induce neuro-inflammation not only in the brain but also in the cochlea [26]. LPS causes damages of the cochlear blood–labyrinth barrier through activating resident macrophages [27], leading to subsequent sensorineural hearing loss in mice [28] and humans [8]. Therefore, we hypothesized that prenatal LPS exposure induces animal models of mood disorders, behavioural alterations, and anxiety-like behaviour, as well as sensorineural hearing loss in offspring. Additionally, dysregulated neural differentiation during embryonic neural development may play a role in the pathophysiology of adverse neurological outcomes.

In the present study, we simulated common scenarios of prenatal infection using FVB mice as well as two mouse ESC lines: the Sox1-green fluorescent protein knock-in ESC line 46C and the ESC line E14Tg2a [29, 30]. The ESCs were treated with LPS, which can bind and activate the downstream signalling cascade of TLR4. We investigated the in vivo effects of this treatment on gestation days (GDs) 9 and 16 on the offspring of pregnant mice using gene expression assays, immunohistochemical staining, and behavioural tests. Moreover, we examined the dynamic changes in the percentage of TLR2^+^ and TLR4^+^ cells during the neural differentiation of the ESC lines in vitro. We observed that differential LPS exposure during the ESC–NSC and NSC–neural differentiation transitions induced varying effects on neuronal and glial differentiation. Our data suggest that the expression profile of TLRs during early neural development and the timing of prenatal infection are both major determinants of the types and severity of adult-onset neural disorders. Dysregulated neural differentiation during embryonic neural development after prenatal infection may be associated with the development of anxiety-like behaviour in adulthood.

## Materials and methods

### Animals

Eight-week-old pregnant FVB/NCrlBltw and C57BL/6 mice were purchased from BioLASCO Taiwan (Taipei, Taiwan) [31–33]. To prevent additional stress for newly arrived animals and to ensure that transportation stress on experimental animals does not confound the data, 6-day pregnant FVB/NCrlBltw mice underwent at least 3-day acclimation period prior to LPS injection. Pregnant females were individually housed in PVC cages under a 12-h light/12-h dark cycle (lights off: 7 PM) with ad libitum access to water and food. All animal studies were conducted in accordance with the animal protocol approved by the MacKay Memorial Hospital (MMH-A-S-106-52). Prenatal challenge was administered through a single intraperitoneal (i.p.) injection of 25 µg/kg LPS on GD 9 or 16. Vehicle control pregnant mice received phosphate-buffered saline (PBS) through i.p. injection on GD 9 or 16. All behavioural tests were evaluated in the male and female offspring from three pregnant dams. Male and female offspring were housed in different cages in the same room. The mice were weaned 3 weeks after birth. The pups of each litter size were housed in groups of four mice per cage (separated by sex), with ad libitum access to water and food. The dose effects of LPS on abortion and offspring survival in the pregnant mice were evaluated. At the age of 8 weeks, the body weights of the PBS-treated control and LPS-treated offspring were recorded, and behavioural tests were subsequently performed.

### Behavioural tests

**Fig. 1:**
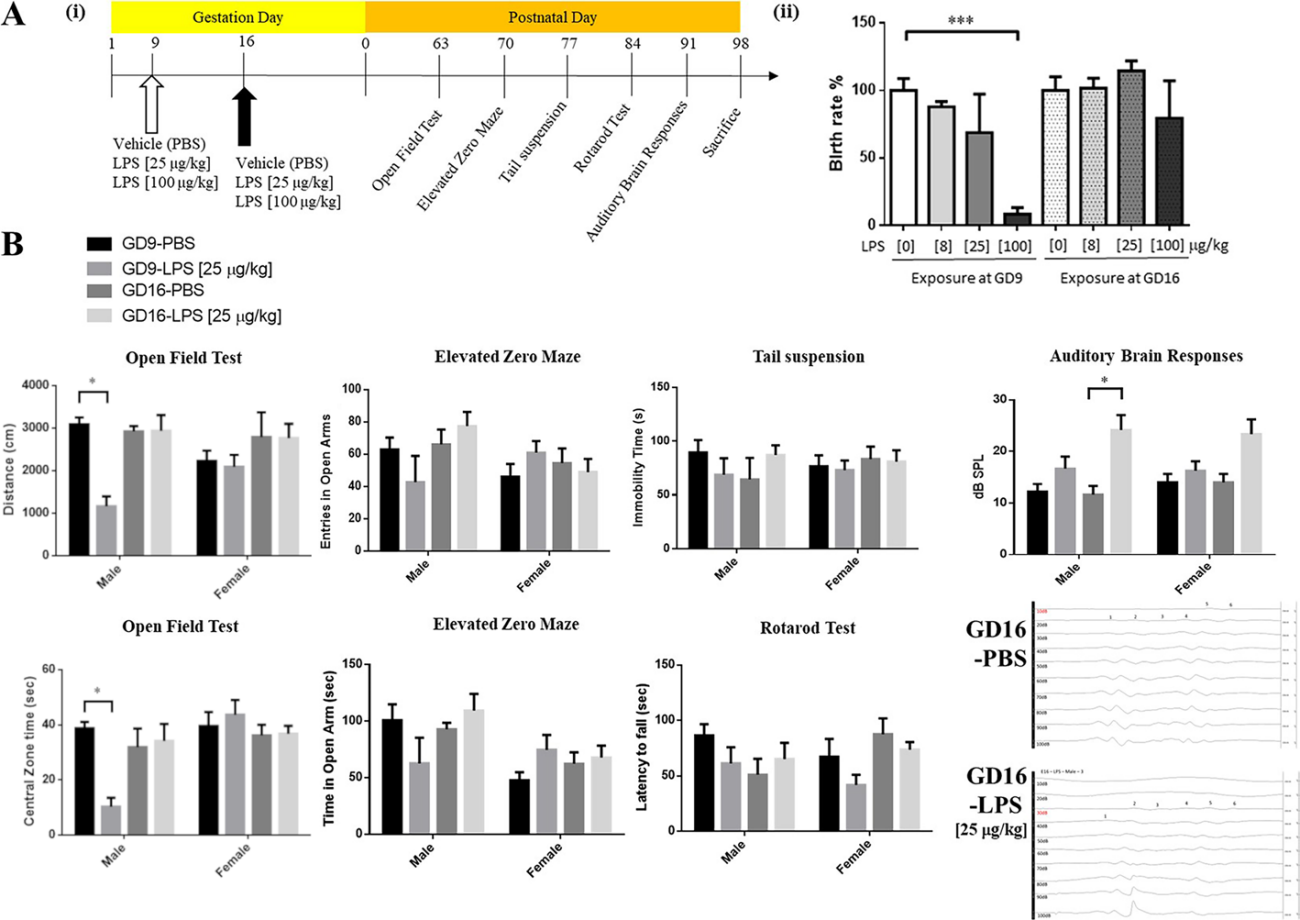
Effects of prenatal exposure to LPS on pregnant mice and their offspring. **A-i** Schematic of the timeline of the experimental design (GD, gestation day). **A-ii** Birth rates of the offspring of pregnant mice with prenatal exposure to different LPS doses. Data are presented as means ± standard error mean (SEM). ***P < 0.0001 versus PBS exposure on GD 9 by one-way ANOVA with the Tukey’s multiple comparisons test. **B** Open filed test: Prenatal exposure to 25 µg/kg LPS on GD 9 significantly decreased the total distance travelled of the male offspring, indicating hypoactivity. Data are presented as means ± SEM. *P < 0.05 versus vehicle control by two-way ANOVA with the Tukey’s multiple comparisons test. Prenatal exposure to 25 µg/kg LPS on GD 9 significantly decreased the total distance of the male offspring. Prenatal exposure to 25 µg/kg LPS on GD 9 or 16 did not affect open arm time of the male offspring. Elevated zero maze: Prenatal exposure to 25 µg/kg LPS on GD 9 or 16 did not affect the time and entries of open arm of the male offspring. Tail suspension: Prenatal exposure to 25 µg/kg LPS on GD 9 or 16 did not affect the immobility time of the male offspring. Rotarod test: Prenatal exposure to 25 µg/kg LPS on GD 9 or 16 did not affect the latency to fall of the male offspring. Auditory brain response: Representative data of auditory brain responses of the male offspring of pregnant mice exposed to 25 µg/kg LPS on GD 9 or 16. Prenatal exposure to 25 µg/kg LPS on GD 16 significantly increased the hearing threshold of the male offspring. Data are presented as means ± SEM. *P < 0.05 versus vehicle control by two-way ANOVA with the Tukey’s multiple comparisons test. In addition, prenatal exposure to LPS did not affect the open field test times, central zone times, open arm times, or number of open arm entries, the latency to fall and auditory brain response of the female offspring

The timeline of the experimental design and behavioural tests is shown in Fig. 1A. All offspring used in the behavioural tests were 8 weeks old, and they were acclimated to the experimental conditions for at least a week before the tests to minimise the effect of experimental manipulation. All mice were weighed, handled, and habituated every day for a week before the tests. Previous studies have demonstrated the importance of handling, acclimatization, and habituation of mice to experimental environments prior to behavioural tests. Such acclimatization can minimize the initial uncertainty when mice are exposed to a novel testing environment [34, 35].

### Open field test

It has been shown that the open field test can be utilized to assess locomotor activity, exploration, and anxiety levels of animals when they encounter a new environment [36, 37]. To familiarize the experimental mice to the sound and light levels of the testing room, all mice in the breeding cages were moved to the testing room 15 min before testing. During the acclimatizing period, 4–5 mice were placed in the breeding cage (dimensions, 44 cm × 22.5 cm × 22.5 cm; illumination, less than 25 lux) for 5 min, not in the testing cage. The brightness of the acclimatizing area was adjusted to match the conditions in the breeding room [38]. The total distance travelled and the time spent in the central zone were recorded using a video camera and analysed using SMART software.

Mice were placed into a white PVC box (dimensions, 40 × 40 × 40 cm; illumination, less than 25 Lux) facing the wall. Total distance travelled was analysed in 5-min intervals. We recorded videos for subsequent analysis of risk assessment using SMART software, and an experimenter blind to the experimental conditions assessed rearing behaviour. The temperature of the behavioural testing room was maintained at 25–28 °C. It is crucial to ensure that the corners of the box are not significantly darker to prevent bias in the activity of the animal. Accordingly, we used evenly distributed light (indirect lighting) and measured illumination with a lux meter to maintain the illuminance below 25 lux. The behaviours of male and female mice were assessed on different days in different testing cages to avoid the influences of sex hormones on mouse behaviours. The tests were performed during the first half of the lights-on period, specifically in the morning until early afternoon, from 10 AM to 2 PM.

### Elevated zero maze

The elevated zero maze (EZM) consisted of a ring-shaped apparatus (diameter, 50 cm) with two open and two closed arms (dimensions, 5 cm × 16 cm) placed 50 cm above the ground and illuminated with low light (below 25 lux). The mice were placed on the wall area, facing an open area, and allowed free exploration for 5 min [39]. The time spent in the area of open arms and the number of entries into the area of open arms were analysed using CLEVER (CleverSys, VA, USA).

### Tail suspension test

To test depression-like behaviour, we suspended each mouse in an acrylic box (dimensions, 11.5 cm × 60 cm; height, 50 cm) from a hook attached to an acrylic gauge by its tail, using acrylic tape positioned 2 cm from the tip of its tail. The distance from the mouse’s nose to the ground was 30 cm [40]. The suspended mouse was recorded using a video camera for a total of 6 min, and the immobility time during suspension was calculated.

### Rotarod test

The locomotor ability of mice was evaluated on a rotarod with a diameter of 3 cm. We followed the protocol described by Zhou et al. [41]. The experimental protocol consisted of two phases: habituation (day 1) and rotarod training/testing (days 2–5). During the habituation phase on day 1, the mice were trained to maintain their position on the rotarod while the rotarod rotated at a constant speed of 3 rpm. This phase allowed the mice to become accustomed to the apparatus and the task. The training/testing phase took place from days 2 to 5. In each test, mice were placed on the rotarod rotating at a consistent speed and underwent three 1-min trials. There was a 5-min rest interval provided between each trial. On each test day, the speed of the rotarod was progressively increased, reaching a maximum of 33 rpm by day 5. The primary measurement was the length of time the mice remained on the rotarod without falling off during each trial.

### Auditory brainstem response

To assess the hearing threshold of the offspring from the LPS-exposed dams, an auditory brainstem response instrument (Biopac Systems, Goleta, CA, USA) was used. Mice were anaesthetized using Zoletil (40 mg/kg)–xylazine (9.3 mg/kg; i.p.). Brainwaves I–V were recorded from the scalp using a subdermal needle electrode placed at three locations: (1) the vertex (+), (2) below the pinna of the left ear (−), and (3) under the skin of the back (ground). Stimuli were delivered as 12 kHz clicks for 100 ms, with a sound intensity range of 10 to 100 dB. For detailed information, please refer to our previous publication [42].

### NanoString gene expression analysis

Total mRNA was extracted from the prefrontal cortex, striatum, or hippocampus tissues of each group using a Total RNA Extraction Kit (ThermoFisher Scientific, MA, USA). The extracted mRNA was used to analyse the genes involved in the neuro-inflammation processes on a NanoString mouse neuropathology panel (NanoString Technologies, Seattle, WA, USA) at Cold Spring Biotech (Taiwan). The gene expression values are presented as percentages of the control. The gene and pathway scores were calculated following the user manual of nCounter Advanced Analysis Software and as described previously [43, 44]. Briefly, we analysed the expression levels of genes involved in various biological pathways and characteristics of different cell types using the NanoString neuropathological gene expression panel. The results obtained from pathway score or cell type-specific score analyses complement those obtained through a more gene-focused approach. Gene or pathway scores were used to condense the data from a pathway-specific or cell type-specific genes into a single score by using nCounter® Advanced Analysis Software (version 2.0.134; https://www.nanostring.com/products/analysis-software/advanced-analysis). These scores were calculated as the first principal component of the normalized expression of the pathway genes.

### Immunohistochemical staining

For the euthanasia procedure, we used excessive CO_2_ on postnatal day 98. Mice were exposed to excessive CO_2_ until they became breathless and then perfused from the heart. We used PBS to flush the blood, 4% PFA as a fixation solution, and severed the head to remove the brain. The brain was soaked in 4% PFA overnight, dehydrated using 15% and 30% sucrose in sequential order, and frozen and embedded with OCT cryostat sectioning medium. Frozen brain sections, specifically those from the prefrontal cortex, striatum, and hippocampus, were placed in 10% formalin overnight for post-fixing. Then, they were immersed in 15% sucrose and, subsequently, 30% sucrose for dehydration. The brain slices were stained with primary antibodies against MAP2 (1:200; Millipore), TuJ1 (1:200; Millipore), GFAP (1:250; Novus), O4 (1:200; R and D Systems), and IBA1 (1:200; Novus). Subsequently, the secondary antibodies goat CY3 (1:400; Millipore), mouse Alexa 594 (1:400; Jackson Immuno Research), and rabbit Alexa 488 (1:400; Jackson Immuno Research) were applied. The immunohistochemical staining images were captured using a confocal microscope (Leica SP8).

### Western blot analysis

Forty micrograms of total protein were used for western blot analysis, as described previously [40]. Briefly, total protein lysates were separated on a 10% SDS–PAGE and transferred onto Immobilon-PVDF (Millipore). Blots were incubated with primary antibodies against MAP2 (1:200, Millipore), GFAP (1:1000, Novusbio), CNPase (1:1000, Genetex), and GAPDH (1:2000 for Western blot, Genetex). Primary antibodies were diluted in Tris-buffered saline Tween 20 (TBS-T) containing 5% milk and 0.01% sodium azide. After antibody incubation, the blots were washed with TBS-T for 1 h and incubated with anti-goat IgG conjugated with horseradish peroxidase (HRP) (Santa Cruz Biotechnology) for 1 h at room temperature. Protein bands were detected by using a chemiluminescent HRP substrate (Millipore) and X-ray films. ImageJ software was used for densitometric analysis. In this study, NanoString gene expression analysis, western blot analyses, and other analyses were performed using samples from male offspring that underwent behavioural tests.

### ESC culture and serum-free embryonic body differentiation

Mouse feeder-free 46C ESCs [29, 30] were provided by Austin Smith (University of Cambridge, UK). The parental ESCs (E14Tg2a.41 cells) were cultured [29, 30] and differentiated [45] as described previously. Cell viability was analysed using 3-[4,5-dimethylthiazol-2-yl]-2,5 diphenyltetrazolium bromide (MTT) assay, as described previously [46].

### Neurosphere assay

Neurosphere assay was performed as described previously [47, 48]. Briefly, we plated the ES cells or ES-derived NSCs (10^4^ cells/mL) on low attachment culture dish in serum-free medium with 20 ng/mL fibroblast growth factor 2 (FGF2), 2 µg/mL heparin and 20 ng/mL epidermal growth factor (EGF; Sigma).

### Reverse transcription and quantitative polymerase chain reaction

Total RNA was isolated using an RNA clean-up kit (FavorPrep, WA, Australia), and cDNA was synthesized using a cDNA synthesis kit (Bio-Rad, CA, USA) following the manufacturers’ instructions. Quantitative polymerase chain reaction (qPCR) was performed using the KAPA SYBR FAST qPCR kit (Kapa Biosystems, Cape Town, South Africa) on a 7900HT fast real-time PCR system. The primers used in qPCR are listed in Additional file 1: Table S1.

### Flow cytometry analysis for TLR2^+^ and TLR4^+^ cells

The cells were trypsinized, resuspended in PBS and analysed using a flow cytometer (FACSCalibur; BD Biosciences, Franklin Lakes, NJ, USA). The cells were stained with anti-TLR2-PE antibody (BD Biosciences) or anti-TLR4-PE antibody (eBioscience, San Diego, CA, USA) before the analysis.

### Immunofluorescent staining

Cells were fixed with 4% paraformaldehyde at room temperature for 15 min and treated in 0.2% Triton X-100 and 1% bovine serum albumin for 1 h for blocking against non-specific binding and permeabilization. The cells were then incubated overnight with a primary antibody, such as anti-MAP2, anti-GFAP or anti-O4, at 4 °C. Thereafter, fluorescein isothiocyanate (FITC)-conjugated and tetramethyl rhodamine-conjugated specific secondary antibodies (1:500; Invitrogen, Carlsbad, CA, USA) were used to detect the primary antibody. The images were captured using a Leica SP8 confocal fluorescence microscope.

### Statistical analyses

The results were expressed as the mean ± standard error of the mean (SEM). Two-way ANOVA was performed with sex and LPS exposure as variables. Tukey’s post hoc test was used to compare the data from multiple groups in mouse behaviour experiments and western blot analyses of MAP2, GFAP, and O4 in different brain regions. One-way ANOVA with Tukey’s post hoc test was used to compare the data from multiple groups in the analyses of birth rate of pregnant mice with prenatal LPS exposure, neurosphere formation, flow cytometry and the quantitative PCR. Differences with *P* < 0.05 were considered significant.

## Results

### Effects on the offspring of pregnant mice exposed to LPS

We first investigated the effects of different doses of LPS on the offspring of pregnant mice. A low (8 or 25 μg/kg) or high (100 μg/kg) dose of LPS was administered intraperitoneally once on GD 9 or 16 to pregnant mice (F_7,31_ = 7.974, *P* < 0.0001). In the GD 9 group, the pregnant mice exhibited a significantly lower birth rate after exposure to 100 μg/kg LPS (9 ± 4.7%, *P* < 0.0001) compared with those exposed to vehicle (PBS; 100 ± 8.9%). The birth rates of the pregnant mice receiving 8 μg/kg and 25 μg/kg LPS were 88 ± 3.8% and 69 ± 28.5%, respectively. In particular, the abortion rate was relatively high in the mice exposed to 100 μg/kg LPS on GD 9, with only one male offspring surviving (in Fig. 1A-ii). Thus, 25 μg/kg LPS was used in the subsequent experiments. The experimental protocol is illustrated in Fig. 1A-i. From 8 to 11 weeks of age, the body weights of the offspring were not significantly different among the different LPS exposure groups (Additional file 1: Fig. S1). Notably, the group of LPS exposure on GD 9 demonstrated sex-dependent differences in the open field test (*F*_3,81_ = 4.184, *P* = 0.0083; Fig. 1B). The male offspring of the pregnant mice exposed to LPS on GD 9 demonstrated hypoactivity in the open field test compared with the controls (PBS group, 3092 ± 157.9, *n* = 10; LPS group, 1160 ± 234.2, *n* = 7; *P* < 0.05), but this was not the case for the GD 16 group (PBS group, 2921 ± 123.8, *n* = 13; LPS group, 2935 ± 367.9, *n* = 13). On the basis of the central zone time (*F*_3,81_ = 4.507, *P* = 0.0056), the male offspring of the pregnant mice exposed to LPS on GD 9 exhibited anxiety-like behaviour (PBS group, 38 ± 2.3, *n* = 10; LPS group, 10 ± 3.1, *n* = 7; *P* < 0.05), but not the female offspring of the pregnant mice exposed to LPS on GD 9 (PBS group, 40 ± 5.1, *n* = 13; LPS group, 43 ± 5.3, *n* = 13) or GD 16 (PBS group, 36 ± 3.8, *n* = 14; LPS group, 37 ± 2.8, *n* = 13), or the male offspring of the mice exposed to LPS on GD 16 (PBS group, 31 ± 6.7, *n* = 6; LPS group, 34 ± 6.1, *n* = 13) (Fig. 1B).

In the EZM experiment (*F*_3,81_ = 2.219, *P* = 0.0921), a lower (though not significant) number of entrances to open arms was observed in the male offspring of the pregnant mice exposed to LPS on GD 9 (PBS group, 62 ± 7.4, *n* = 10; LPS group, 42 ± 16, *n* = 7; *P* = 0.87; Fig. 1B). In addition, a shorter time period spent in open arms was observed in the male offspring of the pregnant mice exposed to LPS on GD 9 (*F*_3,81_ = 2.208, *P* = 0.0935), although the difference was not significant (PBS group, 100 ± 14.1, *n* = 10; LPS group, 62 ± 22, *n* = 7; *P* = 0.6; Fig. 1B).

To further assess the depression-like behaviour of the mice, we analysed immobility by performing a tail suspension test (*F*_3,81_ = 0.6389, *P* = 0.5921). The data suggested no significant differences between the LPS and control groups in males (GD 9: PBS, 89 ± 11, *n* = 10; LPS, 68 ± 15, *n* = 7; *P* = 0.95. GD 16: PBS, 64 ± 20, *n* = 6; LPS, 87 ± 9.0, *n* = 13; *P* = 0.92) or in females (GD 9: PBS, 76 ± 10, *n* = 13; LPS, 73 ± 8.8, *n* = 13; *P* > 0.99. GD 16: PBS, 83 ± 11, *n* = 14; LPS, 80 ± 10, *n* = 13; *P* > 0.99) (Fig. 1B).

In the rotarod experiment (*F*_3,41_ = 1.040, *P* = 0.3850), a shorter (though not significant) time of latency to fall was observed in the male offspring of the pregnant mice exposed to LPS on GD 9 (PBS group, 86 ± 9.9, *n* = 6; LPS group, 61 ± 15, *n* = 6; *P* = 0.0893).

Auditory brain responses in the offspring (*F*_3,68_ = 10.74, *P* < 0.0001) indicated a significantly elevated hearing threshold in the male offspring in the LPS group on GD 16 (PBS, 11 ± 1.6, *n* = 6; LPS, 24 ± 2.8, *n* = 12; *P* < 0.05) but not on GD 9 (PBS, 12 ± 1.4, *n* = 9; LPS, 16 ± 2.3, *n* = 9; Fig. 1B). There was no significant difference among the female offspring on GD 9 (LPS group, 16 ± 1.8, *n* = 8; PBS group, 14 ± 1.6, *n* = 10) and GD 16 (LPS group, 23 ± 2.8, *n* = 12; PBS, 14 ± 1.6, *n* = 10) (Fig. 1B).

**Fig. 2:**
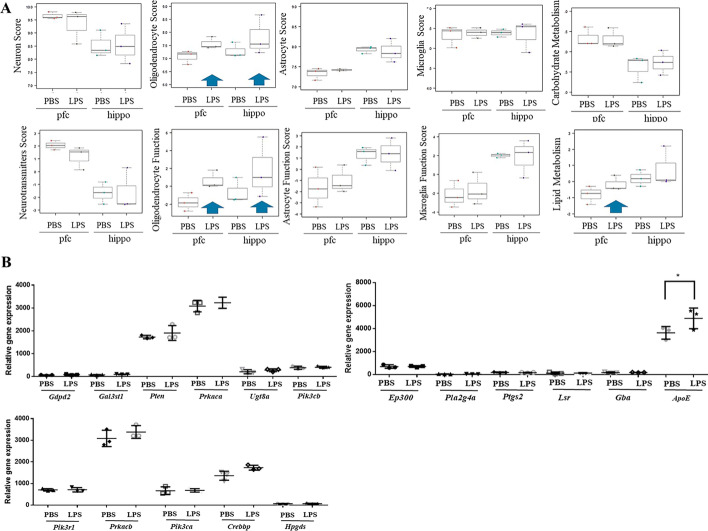
NanoString gene expression analyses of the brain tissues from the male offspring of pregnant mice exposed to LPS. **A** After prenatal exposure to 25 µg/kg LPS, the gene scores for neurotransmissions slightly decreased and those for oligodendrocytes and oligodendrocyte function increased in the prefrontal cortex (pfc) and hippocampus (hippo) tissues of the male offspring. Lipid metabolism gene scores increased in the tissues from the prefrontal cortex of the male offspring but not in those from their hippocampus after prenatal exposure to 25 µg/kg LPS. The gene scores of neurons, neurotransmitters, astrocytes, astrocyte function, oligodendrocytes and oligodendrocyte function, microglia, microglia function, carbohydrate metabolism and lipid metabolism in the prefrontal cortex and hippocampus tissues of the male offspring were affected by prenatal exposure to 25 µg/kg LPS. **B** mRNA expression of selected genes involved in lipid metabolism in the NanoString gene profiling assay. Data are presented as means ± SEM. * P < 0.05 versus 0 µg/kg LPS by one-way ANOVA with the Tukey’s multiple comparisons test

**Fig. 3:**
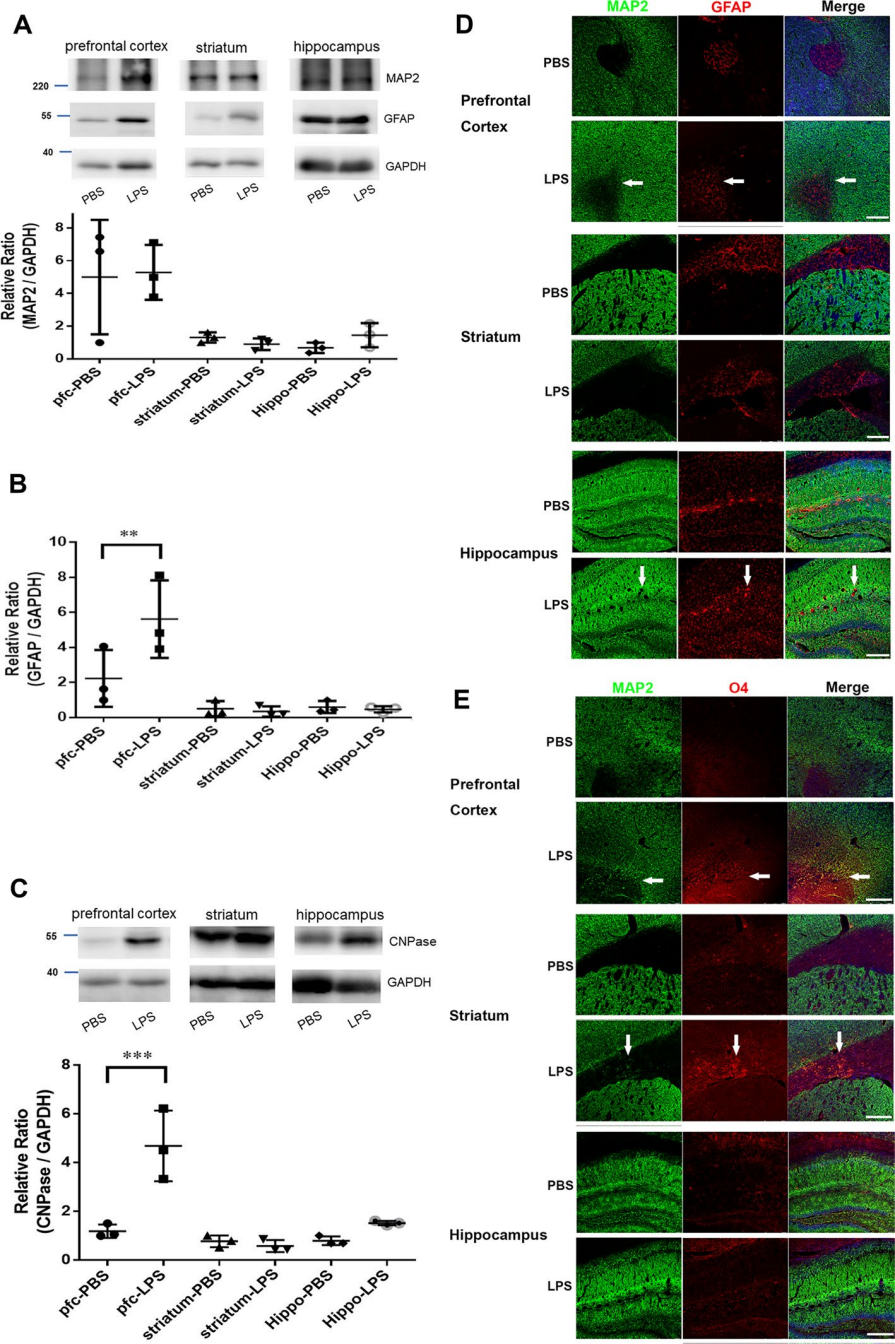
Western blot and immunohistochemical analyses of the neurons, astrocytes, and oligodendrocytes in different brain regions of the male offspring after prenatal LPS exposure. Representative western blots for the **A** neuronal markers MAP2, GFAP; semi-quantification of MAP2/GAPDH; **B** semi-quantification of GFAP/GAPDH, and **C** oligodendrocyte marker CNPase and semi-quantification of CNPase/GAPDH in the prefrontal cortex, striatum, and hippocampus tissues of the male offspring after prenatal exposure to 25 µg/kg LPS. Statistical analyses from three representative samples of western blot for the neuronal marker MAP2 are shown in the lower panel. Data are presented as means ± SEM. ** P < 0.01 versus PBS group by one-way ANOVA with the Tukey’s multiple comparisons test. **D** Representative images of immunohistochemical double staining for MAP2^+^ and GFAP^+^ cells in the prefrontal cortex, striatum and hippocampus tissues. GFAP^+^ cells were more abundant in the prefrontal cortex and hippocampus tissues of the male offspring after prenatal exposure to 25 µg/kg LPS on GD 9. **E** Representative images of immunohistochemical double staining for MAP2^+^ and O4^+^ cells in the prefrontal cortex, striatum, and hippocampus tissues. O4^+^ cells were more abundant in the prefrontal cortex and hippocampus tissues of the male offspring after prenatal exposure to 25 µg/kg LPS on GD 9. Scale bar, 250 µm. Arrows indicate GFAP^+^ or O4^+^ cell morphology

In the NanoString gene expression profiling analyses, the gene scores of the neurotransmitters in the prefrontal cortex and hippocampus tissues were slightly downregulated in the male offspring of the pregnant mice exposed to LPS on GD 9 (Fig. 2A). Notably, in the male offspring of the pregnant mice exposed to LPS on GD 9, the gene scores for the oligodendrocytes and the oligodendrocyte function in the prefrontal cortex and hippocampus tissues were upregulated (Fig. 2A, indicated by blue arrows). In addition, the gene scores for the astrocytes and microglia demonstrated no changes among prefrontal cortex and hippocampus tissues in the male offspring of the pregnant mice exposed to LPS on GD 9 (Fig. 2A). We performed western blot and immunohistochemistry analyses by using cell type-specific antibodies, such as those against MAP2 for neurons [degrees of freedom (DF) = 2, *F* = 0.1975, *P* = 0.8234; Fig. 3A, D, E ], GFAP for astrocytes (DF = 2, *F* = 11.34, *P* = 0.0017; Fig. 3A, B, D), O4 for oligodendrocytes (DF = 2, *F* = 11.39, *P* = 0.0017; Fig. 3C, E), CNPase for oligodendrocytes, and IBA1 for microglia. In the prefrontal cortex region of the male offspring of the pregnant mice exposed to LPS on GD 9, we observed significantly increased numbers of GFAP^+^ astrocytes (*P* = 0.0012; Fig. 3B) and CNPase^+^ and O4^+^ oligodendrocytes (*P* = 0.0003; Fig. 3C, E). However, we did not observe any changes in the IBA1^+^ microglia in the prefrontal cortex, striatum, or hippocampus (Additional file 1: Fig. S2). In addition, in the prefrontal cortex tissues of the male offspring of the pregnant mice exposed to LPS on GD 9, the gene score for lipid metabolism was abnormally high (Fig. 2A, blue arrow), indicating a correlation with an aberrant increase in astrocyte and oligodendrocyte differentiation in the prefrontal cortex and hippocampus tissues of these mice. The genes included in the lipid metabolism score calculations were *Gdpd2*, *Gal3st1*, *Pten*, *Prkaca*, *Ugt8a*, *Pik3cb*, *Ep300*, *Pla2g4a*, *Ptgs2*, *Lsr*, *Gba*, *ApoE*, *Pik3r1*, *Prkacb*, *Pik3ca*, *Crebbp*, and *Hpgdsg* (Fig. 2B). Notably, the relative gene expression of *ApoE* was significantly higher in the prefrontal cortex tissues of the male offspring of the pregnant mice exposed to LPS on GD 9 compared with the PBS vehicle group (*P* < 0.0001, *n* = 3; Fig. 2B).

### Establishment of neural differentiation platform of ESC–NSC transition and NSC–neural differentiation transition using mouse 46C ES cells

**Fig. 4:**
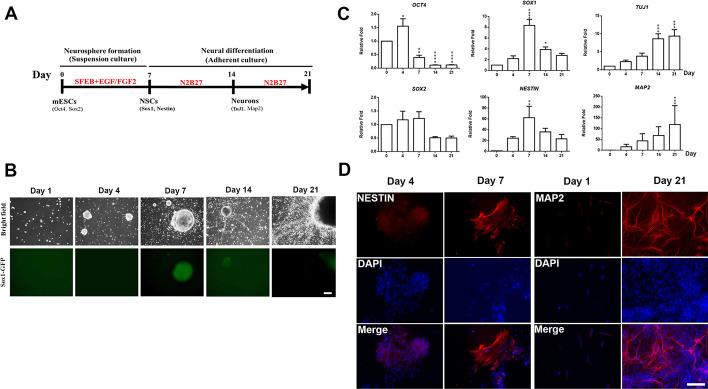
Establishment of neural differentiation platform of mouse embryonic stem cells. **A** Schematic of the ESC-to-NSC and NSC-to-neural differentiation transition processes. **B** Neurosphere formation during ESC-to-NSC transition and NSC-to-neural differentiation transition in 46C ES cells. Neurosphere size and number increased from day 1 to days 4 and 7. Scale bar, 100 µm. **C** qPCR for mRNA expression of ESC markers (*Oct4* and *Sox2*), NSC markers (*Sox1* and *Nestin*) and neuronal markers (*Tuj1* and *Map2*). Data are presented as means ± SEM (*n* = 3). Significant difference and *P* values were analysed by one-way ANOVA with the Tukey’s multiple comparisons test. **D** Immunocytochemical analyses for the neural stem cell marker NESTIN and the neuronal marker MAP2 after neural differentiation for 14 and 21 days. Scale bar , 100 µm

LPS exposure on GD 9 resulted in male offspring exhibiting anxiety-like behaviour, which might be caused by disturbances in ESC–NSC transitions during foetal brain development. An in vitro cell model was used to further clarify the underlying mechanism. We established a neural differentiation platform using mouse ESC lines to model prenatal infection during embryonic development by using 46C ES cells and the neural differentiation protocol developed by Smith et al. [29, 30]. A schematic of the neural differentiation platform of mouse ESCs is depicted in Fig. 4A. We observed green fluorescence in NSCs derived from 46C ES cells on day 7, which gradually decreased during the NSC–neural differentiation transition (Fig. 4B), consistent with the findings of Smith et al. [29, 30]. Next, we used quantitative PCR (qPCR) to measure the mRNA expression of the ESC (*Oct4* and *Sox2*), NSC (*Sox1* and *Nestin*), and neuronal (*Tuj1* and *Map2*) marker genes during ESC neural differentiation on days 0 (ESC stage), 4, 7 (NSC stage), 14 and 21 (differentiated neural progenies). *Oct4* mRNA expression was abundant in the ESC stages (relative fold change, 1.55 ± 0.27; ESC stage day 7 versus 0; *P* < 0.05) but not in NSC or neural progeny stages differentiated from mouse ESCs [7]. In addition, *Sox2* was predominantly expressed in the ESC [45] and NSC [46] stages (relative fold change, 1.17 ± 0.3; ESC stage day 7 versus 0; Fig. 4C). Here, differences in *Sox1* (relative fold change, 8.34 ± 1.12; day 7 versus 0; *P* < 0.0001) and *Nestin* (relative fold change, 62.2 ± 21.1; day 7 versus 0; *P* < 0.001) mRNA expression levels were detected in the NSC stage after ESC differentiation for 7 days. Notably, *Sox1* and *Nestin* mRNA levels gradually decreased during the NSC–neural differentiation transition (Fig. 4C). The mRNA levels of the neuronal markers *Tuj1* (relative fold change, 9.37 ± 1.80; day 21 versus 0; *P* < 0.001) and *Map2* (relative fold change, 119.3 ± 33.0; day 7 versus 0; *P* < 0.001) were gradually and significantly upregulated during the ESC–NSC transition (days 0–7) and NSC–neural differentiation transition (days 7–14 or days 7–21; Fig. 4C). Immunofluorescence staining results indicated that the NSC-differentiated neural progenies also demonstrated *Tuj1* and *Map2* expressions on days 14 and 21 (Fig. 4D).

### Percentages of TLR2^+^ and TLR4^+^ cells during ESC–NSC transition

**Fig. 5:**
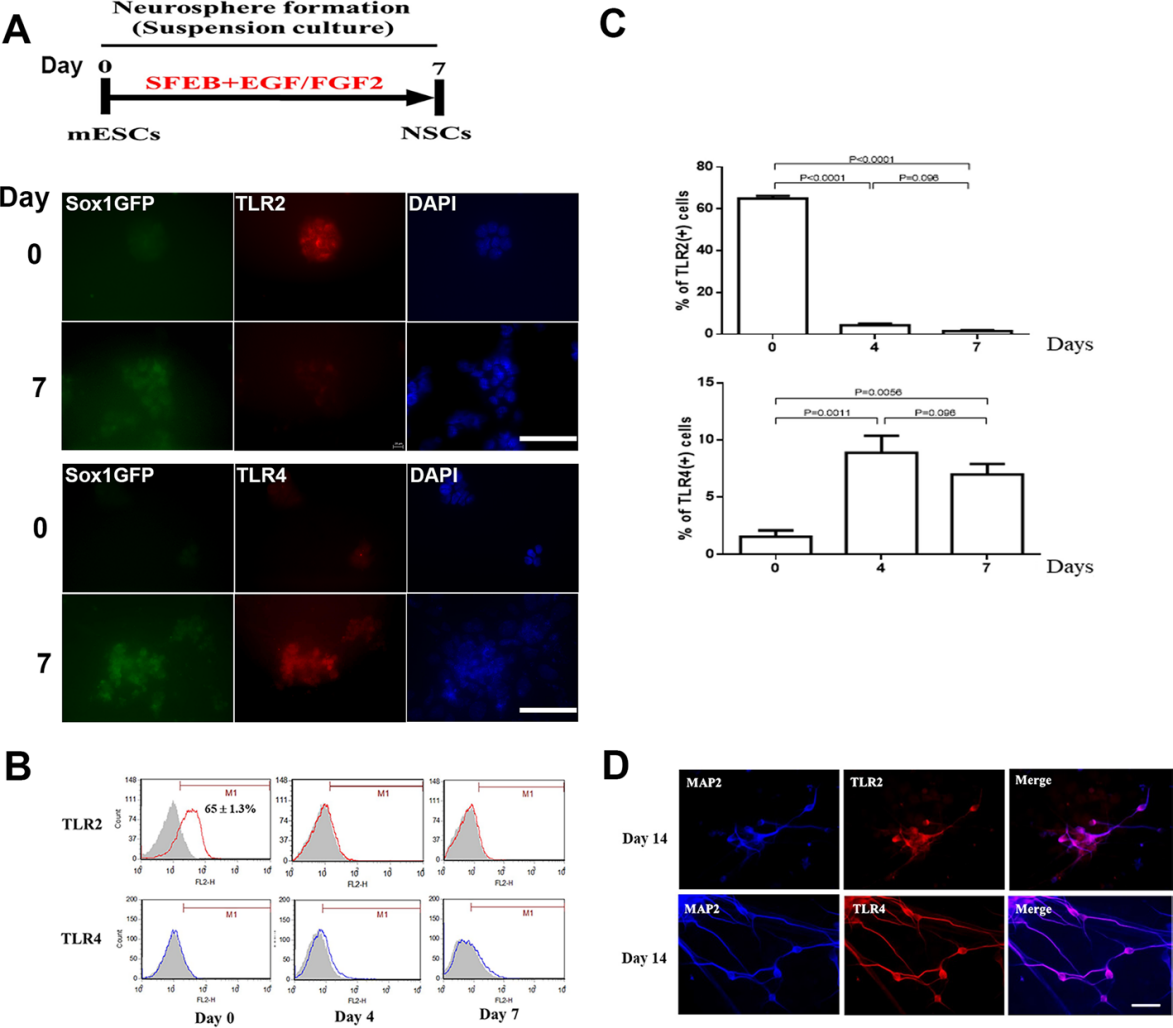
Expression of toll-like receptor 2 (TLR2) and 4 (TLR4) during ESC-to-NSC transition and NSC-to-neural differentiation transition processes. **A** Schematic of the ESC-to-NSC transition process. Immunofluorescence staining for TLR2 and TLR4 expression in the differentiated cells on days 0 and 7 during ESC-to-NSC transition. Scale bar, 100 µm. **B**, **C** Quantification of TLR2^+^ and TLR4^+^ cells in ESCs and ESC-derived NSCs. Data are presented as means ± SEM (*n* ≥ 6). Significant difference and *P* values were analysed by one-way ANOVA with the Tukey’s multiple comparisons test. **D** Immunofluorescence staining for TLR2 and TLR4 in the differentiated MAP2^+^ cells on day 14 after NSC differentiation. Scale bar, 100 µm

TLR2 and TLR4, which can be detected on NSCs [18], are involved in neural progenitor cell and NSC proliferation and differentiation [19, 20]. To investigate the effects of LPS during ESC–NSC transitions, we determined the percentages of TLR2^+^ and TLR4^+^ ESCs and ESC-derived NSCs (Fig. 5). Immunofluorescent double staining results demonstrated that TLR2 was strongly expressed in the ESCs at day 0 (Fig. 5A). On day 7, 46C ES cells also expressed TLR4 (Fig. 5A). We also observed that, during ESC–NSC transitions, the percentage of TLR2^+^ cells gradually and significantly decreased from 65% ± 1.3% to 1.6% ± 0.4%, whereas that of TLR4^+^ cells significantly increased from 1.6% ± 0.5% to 7.0% ± 0.9% (Fig. 5B, C). On day 14, we observed that the MAP2^+^ neurons expressed both TLR2 and TLR4 (Fig. 5D), suggesting that the differentiated neurons expressed TLR2 and TLR4 in situ. We further utilized the parental mouse ESC line, E14Tg2a, to verify the percentages of TLR2^+^ and TLR4^+^ during ESC–NSC transitions, which were similar to those in the 46C ES cells (Additional file 1: Fig. S3).

### Percentages of TLR2^+^ and TLR4^+^ cells in embryonic and adult mouse brains

To examine the correlation between the percentages of TLR2^+^ and TLR4^+^ cells during neural differentiation and development in vitro and in vivo, we analysed the percentages of TLR2+ and TLR4+ cells in embryonic NSCs (E10, E14 and E15), as well as adult (1-month-old and 2-month-old) FVB and C57BL/6 mouse brains. The highest percentages of TLR2^+^ and TLR4^+^ cells were observed in the E10 NSCs (7.2% ± 0.2% and 10.2% ± 0.6%, respectively), followed by the E14 (6.8% ± 0.02% and 7.4% ± 0.4%, respectively) and E15 (3.7% ± 0.3% and 6.9% ± 1.4%, respectively) NSCs (Additional file 1: Fig. S4A). The percentages of TLR2^+^ and TLR4^+^ cells were 5.6% ± 0.9% and 5.6% ± 1.1%, respectively, in the 1-month-old FVB mouse brains, while they were 7.9% ± 1.6% and 9.8% ± 1.1%, respectively, in the 2-month-old FVB mouse brains (Additional file 1: Fig. S4A). Moreover, the percentages of TLR2^+^ and TLR4^+^ cells in the 1-month-old C57BL/6 mouse brains were 6.6% ± 0.9% and 12.0% ± 1.5%, respectively, and in the 2-month-old C57BL/6 mouse brains, they were 6.8% ± 1.9% and 5.8% ± 1.2%, respectively (Additional file 1: Fig. S4B). Based on our data, the relative percentage of TLR4^+^ cells in the adult mouse brains (5–10% of the total cells; Additional file 1: Fig. S4) was similar to that observed in the embryonic NSCs in vitro on day 7 (5–10% of the total cells; Fig. 4). In contrast, the relative percentage of TLR2^+^ cells in the adult mouse brains (5–10% of the total cells; Additional file 1: Fig. S4) was similar to that in the embryonic NSCs in vitro on day 7 (less than 1% of the total cells; Fig. 4). Therefore, our findings suggest that TLR4 may play a significant role as a mediator of LPS signalling in embryonic NSCs during neuro-development.

### Effects of LPS on neurosphere formation during ESC–NSC transition of ESCs and ESC-derived NSCs

**Fig. 6:**
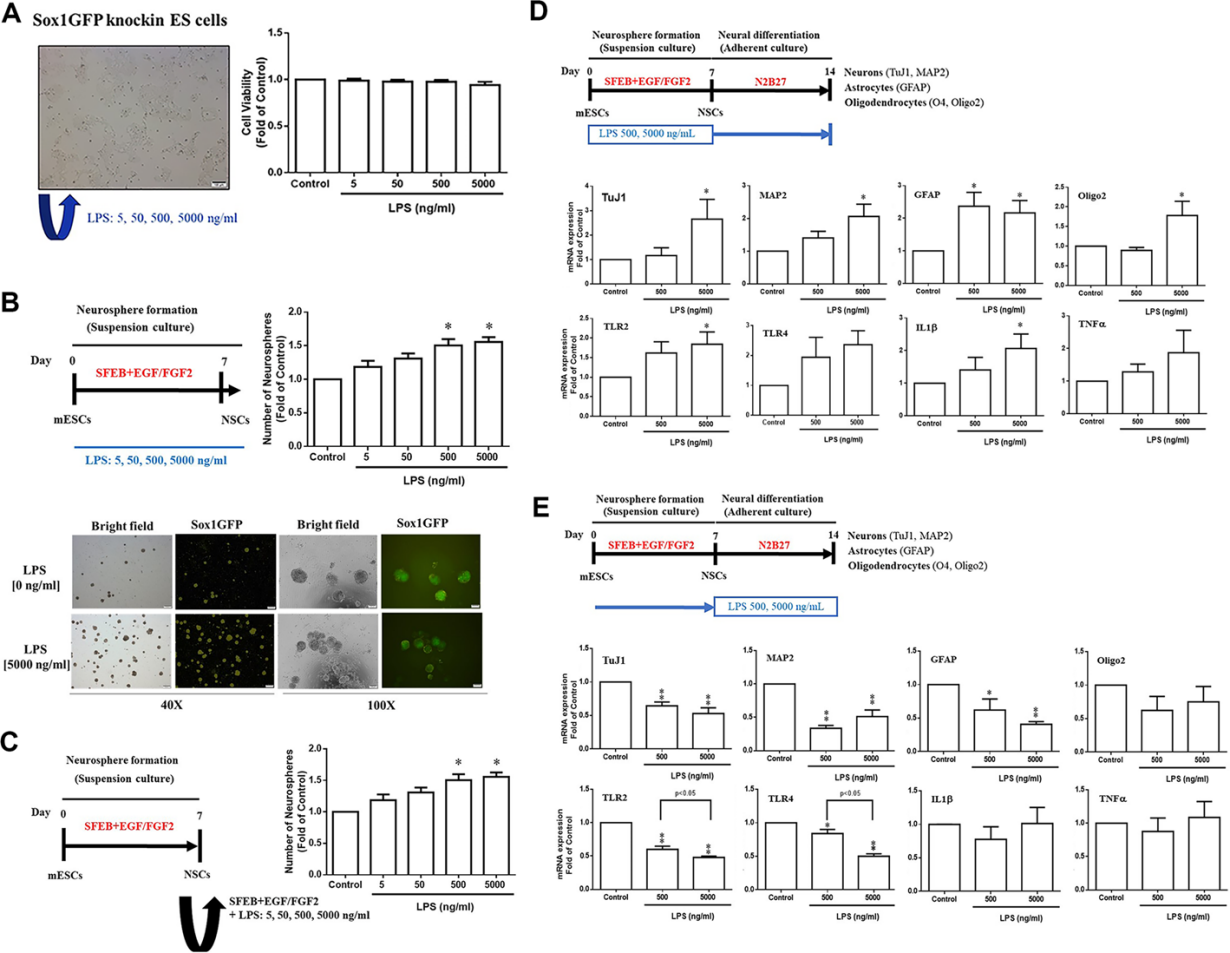
Effects of LPS on neurosphere formation capacities during ESC-to-NSC transition. **A** The different doses of LPS (5, 50, 500 and 5000 ng/mL), supplied through the ESC culture medium, did not affect 46C ES cell viability. Data are presented as means ± SDs (*n* ≥ 3). Significant difference and *P* values were analysed by one-way ANOVA with the Tukey’s multiple comparisons test. **B** Schematic of LPS treatment during ESC-to-NSC transition. Representative images of the effects of LPS treatment on neurosphere formation during ESC-to-NSC transition. Scale bar, 100 µm. Quantification of neurosphere formation after LPS treatment during ESC-to-NSC transition. Data are presented as means ± SEM (*n* ≥ 3). Significant difference and *P* values were analysed by one-way ANOVA with the Tukey’s multiple comparisons test. **C** Schematic of the effects of LPS treatment on self-renewal (neurosphere reformation) capacity of ESC-derived NSCs after treatment with different doses of LPS (5, 50, 500 and 5000 ng/mL). Data are presented as means ± SEM (*n* ≥ 3). Significant difference and *P* values were analysed by one-way ANOVA with the Tukey’s multiple comparisons test. Schematic of effects of LPS (500 or 5000 ng/mL) during **D** ESC-to-NSC transition (days 0–7) and **E** NSC-to-neural differentiation transition (days 7–14) on neural differentiation capacities of ESCs and ESC-derived NSCs; differentiated cells were assessed on day 14. Effects of LPS on the mRNA expression levels of the neuronal markers *Tuj1* and *Map2*; the astrocytic marker *Gfap*; the oligodendrocyte markers *Oligo2*; and inflammatory marker genes *Tlr2*, *Tlr4*, *IL1b* and *Tnfa* after neural differentiation, as evaluated using quantitative PCR. Data are presented as means ± SEM (*n* = 3). *P < 0.05, **P < 0.05 by one-way ANOVA with the Tukey’s multiple comparisons test. SFEB, serum-free embryonic body; N2B27, Dulbecco’s modified Eagle medium supplemented with N2 and B27

We proceeded to examine the impact of LPS on ESC–NSC transitions by utilizing suspension culture of neurospheres. We treated 46C ES cells with various concentrations of LPS (5, 50, 500 and 5000 ng/mL) and observed that LPS did not affect ESC viability (Fig. 6A). Subsequently, we investigated whether LPS would influence ESC–NSC transitions by adding LPS to serum-free embryoid body medium containing EGF and FGF2 (Fig. 6B). Notably, we found that 5000 ng/mL LPS significantly increased the number of neurospheres during the ESC–NSC transition (Fig. 6B). Furthermore, we examined the effects of LPS on the self-renewal (neurosphere reformation) of ES-derived NSCs (Fig. 6C). Similarly, 500 and 5000 ng/mL LPS significantly enhanced their self-renewal capacity by increasing the number of neurospheres (Fig. 6B, C).

### Effects of LPS on neural differentiation capacities of ESCs and ESC-derived NSCs

To investigate the effects of LPS on neural differentiation, we designed two experimental procedures to monitor prenatal infection in vivo (Fig. 6D, E). In the first procedure, LPS was administered to mice during the ESC–NSC transition (days 0–7). The LPS-treated cells were then allowed to differentiate until day 14. At day 14, the mRNA expression levels of the neuronal markers *Tuj1* and *Map2*, the astrocytic marker *Gfap*, and the oligodendrocyte marker *Oligo2* showed significant and dose-dependent upregulation. Concurrently, *IL1b* mRNA expression was also significantly upregulated (Fig. 6D).

In the second procedure, LPS was administered during NSCs–neural differentiation (days 7–14; Fig. 6E). This led to significant downregulation of mRNA expression levels of the neuronal markers *Tuj1* and *Map2*, the astrocytic marker *Gfap*, and the oligodendrocyte markers *Oligo2* and *O4* (Fig. 6E). These results suggest that prenatal infection at different timepoints during neural differentiation and development results in distinct dysregulation in self-renewal and neural differentiation in ESCs and ESC-derived NSCs. Additionally, in whole-transcriptome analyses conducted after the in vitro experiments (Tables 1 and 2), we observed a significant increase in *ApoO* and *ApoB*, which are involved in lipid metabolism and immune response [51], in the differentiated neural cells after LPS treatment during the ESC–NSC transition (Fig. 6D, Table 1).

**Table 1:** Top 20 upregulated genes in neural differentiated cells after LPS treatment during ESC-to-NSC transition (Fig. 6D) and during NSCs-to-neural differentiation transition (Fig. 6E)

Top 20 upregulated genes		
Gene symbol	Real fold changes	PPEE
Amd1	31,330.92888	–
Gm5643	4900.664024	2.7137 × 10^−10^
Dmrtc1c1	4111.674009	1.81877 × 10^−8^
Dcn	4.643054118	0.001902789
Cryaa	4.054753913	4.10895 × 10^−5^
Raet1b	3.922965873	0.000639015
Gm14685	2.790725343	0.000444304
ApoO	2.567679411	0.001653985
Tmem254c	2.525407318	0.006808228
Shisa2	2.501014191	0.002686353
Cp	2.283614759	0.003088226
Dpp4	2.197551207	0.004105459
Pls3	2.179027456	4.78997 × ^−5^
Snurf	2.126767137	0.002332546
Micu2	2.034107028	0.020139765
Shoc2	2.023370502	0.008477638
ApoB	1.960375521	0.033574492
Ddx52	1.941305178	0.01056659
Eif3e	1.939698905	0.001410564

**Table 2:** All downregulated genes in neural differentiated cells after LPS treatment during ESC-to-NSC transition (Fig. 6D) and during NSC-to neural differentiation transition (Fig. 6E)

All downregulated genes		
Gene symbol	Real fold changes	PPEE
Lime1	0.509047096	0.009433626
Ankrd23	0.458260977	0.002080824
1600020E01Rik	0.428361555	0.023253218
Dmrtc1c2	0.356620065	0.010360542
Cd7	0.258265	0.0266615
ApoO-ps	0.239407906	0.002036297
Zfp967	0.041206954	0.000382669
Duxbl1	0.000649415	0.017333353
DXBay18	9.5746 × 10^−5^	–

## Discussion

In this study, we exposed pregnant mice to 25 μg/kg LPS on GD 9 and found that only the male offspring exhibited anxiety-like behaviour at 2 months of age. Through gene profiling analysis, we observed that 2-month-old male offspring of LPS-exposed pregnant mice showed increased numbers and function of oligodendrocytes, as well as enhanced lipid metabolism, in the prefrontal cortex. However, a decrease in neurotransmission activity was detected in both the prefrontal cortex and hippocampus. Moreover, western blot and immunohistochemical analyses revealed significant increases in GFAP and CNPase, as well as significant increase in the numbers of GFAP^+^ and O4^+^ cells, in the prefrontal cortex and striatum regions. Based on these findings, it can be concluded that exposure to LPS on GD 9 induces morphological changes in the prefrontal cortex and striatum, which are essential for regulating anxiety-related behaviour and anxiety states. This is supported by our observation that anxiety-related behaviour was exacerbated by LPS exposure (Fig. 1B). Moreover, the structural, cellular and molecular changes caused by early prenatal infection within the specific regions of the prefrontal cortex and striatum involved in emotional behaviour may contribute to long-term behavioural changes in male offspring, such as heightened anxiety.

Our results also demonstrate potential sex differences in the anxiety-related effects of early prenatal infection in FVB mice (Fig. 1B, C). The 2-month-old male offspring of the LPS-exposed mice entered the open field less frequently compared with the male offspring of vehicle control pregnant mice, whereas female offspring did not exhibit such differences. This result aligns with our finding that LPS exposure resulted in anxiety in male offspring but not in female offspring. Previous studies have demonstrated that sex is associated with distinct behavioural alterations in LPS-treated mice [52, 53]. Additionally, oestradiol has been shown to positively affect cell proliferation [54] and exert neuroprotective effects [55]. Although we did not analyse the level of oestrogen in female mice, it is known to have profound effects on anxiety-like disorders in rodents and humans [56]. Behavioural differences in female offspring are potentially caused by oestrogen; however, discrepancies regarding the nature of oestrogen’s effects on anxiety in preclinical studies and in humans are attributable to the differential effects of specific oestrogen receptor (ER) subtypes [56], indicating the involvement of different genetic backgrounds. Interestingly, a novel type of ER receptor, GPR30, has been reported that its activation through the selective agonist G-1 can decrease anxiety in the open field test but not in the elevated plus maze (EPM) test in female mice, whereas other ER agonist, oestradiol benzoate, had no effect on behaviours in the EPM or the open field [37].

Our in vitro and in vivo studies revealed similar trends to those mentioned above, indicating that early LPS treatment may enhance astrocyte and oligodendrocyte differentiation (Figs. 3, 6). The alterations in oligodendrocytes could play a significant role in mood dysregulation, including anxiety, major depression, and schizophrenia [57–59]. Notably, stress-induced anxiety-like behaviour is positively correlated with hippocampal dentate gyrus oligodendrocytes and myelin basic protein [59]. In addition, overexpression of oligodendrogenic factor *Olig1* through viral infection in the dentate gyrus can induce an anxiety-like behavioural phenotype [57–59]. Furthermore, early weaning may induce anxiety-like behaviour and precocious myelination in certain parts of the amygdala in male Balb/c mice [60]. Our findings suggest that early infection during gestation leads to anxiety-like behaviour in male offspring.

In this study, we observed abnormal behaviour in male offspring of FVB mouse strain exposed to LPS (25 μg/kg) on GD 9, specifically in the open field test. It has been shown that LPS (25 μg/kg) exposure during GD 9 can induce anxiety-like behaviour (as assessed by open field test and EZM) and depression-like behaviour in male C57BL/6 J mice [61], which aligns with our findings on the effects of LPS exposure on behaviours on FVB male offspring. It has also been shown that LPS (25 μg/kg) exposure during GDs 15–17 can lead to the anxiety-like behaviour (open field test and EZM) in female C57BL/6 offspring [62]. Furthermore, in CD1 strain mice, exposure to even lower dose of LPS (8 μg/kg) on GD 9 can result in brain overgrowth and autism-associated behaviours [63]. These findings highlight the influence of genetic backgrounds across different mouse strains, leading to varied effects after LPS exposure on different gestational days. In addition, considering the high genetic effect on anxiety phenotypes is also important for studies to control for litter effects, referring to the fact that pups in the same litter are more phenotypically similar than pups from different litters of the same strain. It has been postulated that there are several major factors that may cause litter effects [64]: (i) social environments across litters—variation in litter size also can influence the developing offspring. According to our data, there was no difference among the number of pups in each litter (Additional file 1: Fig. S1A). (ii) Maternal behaviour on litter effects: according to our data, there was no difference among body weights of male (Additional file 1: Fig. S1B) and female (Additional file 1: Fig. S1C) offspring. (iii) in utero environment: maternal hormones, nutrient levels and environmental chemicals or pollutants passing through the placenta to affect fetus brain development and increase the risk of neural developmental disorders. During pregnancy, infection-associated maternal immune activation can also result in perturbed neural development. From our data, our findings demonstrated that early infection during gestation leads to anxiety-like behaviour in male offspring (Fig. 1) with glial activation in the central auditory system of the offspring from pregnant female mice exposed to LPS (Fig. 2).

Our data suggest the differential effects of prenatal LPS exposure in FVB mouse strain, providing a valuable comparison with previous studies in the literature [61–63].

Furthermore, we observed a significant elevation in the hearing threshold of ABR in male offspring exposed to LPS (25 μg/kg) on GD 16 (Fig. 1B). Our data suggest that prenatal LPS exposure not only induces abnormal behaviour in open field test but also leads to hearing loss. LPS can cause damages of the cochlear blood–labyrinth barrier through the activation of resident macrophages in the coclea [27]. In addition, LPS-injected animals show increased levels of iNOS and O_2_ in the cochlea, leading to sensorineural hearing loss [65]. The elevated hearing threshold may be attributed to abnormal neuro-inflammation in the auditory cortex [66, 67] or the cochlea [26]. Notably, in the tinnitus rats induced by salicylate, a marked increase in GFAP expression is observed in the auditory cortex, accompanied by increased endpoint and process length of astrocyte. Similarly, we found that the expression levels of GFAP and the process length of astrocytes were increased in male offspring of LPS-exposed (GD 9) dams (Fig. 4A, B). These findings highlight the roles of glial activation in the central auditory system of the offspring from pregnant females exposed to LPS.

In the present study, we utilized mouse ESC lines and a neural differentiation platform to model prenatal infection and investigate the mechanisms underlying the development of neural and psychiatric disorders associated with prenatal infection. Although the expression of TLR2 and TLR4 has been demonstrated in adult NSCs in vitro and in vivo, the activation of TLR2 or TLR4 by proinflammatory cytokines does not affect adult NSC proliferation or differentiation [18]. By contrast, TLR2 activation by PAM3 can significantly increase E14Tg2a cell proliferation [68]. Notably, several mouse ESC lines have a high number of TLR2^+^ cells but a lack TLR4^+^ cells [68]. Moreover, mRNA expressions of *Tlr1*, *Tlr2*, *Tlr3*, *Tlr5* and *Tlr6* (but not *Tlr4*) have been detected in E14Tg2a cells [68]. In our study, we observed that the percentages of TLR2^+^ and TLR4^+^ cells in the embryonic and adult mouse brain cells exhibited dynamic changes that are similar to those observed in vitro during the ESC–NSC (corresponding to E3.5–E10.5) and NSC–neural differentiation (corresponding to E10.5–E17.5) transitions. These findings are consistent with those of Kaul et al. [69], as *Tlr2* and *Tlr4* mRNA expression levels remained stable without significant changes in embryonic NSCs (E13–E19), pup brains (P0–P5) and adult mouse brains (5-month-old; Additional file 1: Fig. S4).

Our results suggest that the timing of maternal infection may determine the outcomes of neural disorders in offspring. The expression of TLR2 and TLR4 are transcriptionally upregulated in response to inflammatory exposure. TLR2 and TLR4 expression levels are differentially influenced by the two major proinflammatory cytokines, TNF-α and IFN-γ, respectively. IFN-γ promotes TLR4 induction, whereas TNF-α induces TLR2 expression and counteracts the induction effect of IFN-γ on TLR4 expression. Moreover, TLR2 expression is upregulated after exposure to molecules secreted from activated macrophages. NSCs exposed to TLR2 and TLR4 agonists produce and release TNF-α. Therefore, under neuro-inflammatory conditions, NSCs can upregulate their TLR2 and TLR4 expression levels and respond to receptor activation through the release of proinflammatory cytokines [18]. Only IFN-γ can promote neurite outgrowth during the neuronal differentiation of adult NSCs [70]. Notably, after lysolecithin-induced demyelination, a TLR4 agonist can facilitate remyelination in the white matter of the spinal cord. In vitro, the TLR4 agonist E6020 can induce TNF-α, IL-1β, IL-6 and NF-κB signalling. Microinjection of E6020 into the grey matter–white matter border of an intact spinal cord in rats induces oligodendrocyte progenitor proliferation and differentiation [71].

Embryonic LPS treatment in pregnant dams on embryonic day 9 can induce brain overgrowth, cortical thickening, and a significant increase in Iba1^+^ microglia numbers throughout the brain. It also increases the number of NSCs in the subventricular zone of the offspring on embryonic day 17.5 and postnatal day 0 [63]. Importantly, most offspring exposed to embryonic LPS exhibit abnormal behaviour, such as reduced social and novel behaviours, increased anxiety, and excessive repetitive grooming behaviour on postnatal day 60, which are associated with the effects of brain overgrowth [63]. Our results reveal a significant increase in neurosphere formation capacity on day 7 and in mRNA expression of neuronal and astrocytic markers on day 14, when LPS is administered during the ESC–NSC transition (Fig. 6). These results indicate that early maternal infection during the ESC–NSC transition can enhance the self-renewal and neuronal–astrocytic differentiation capacities of ESCs, potentially contributing to prenatal infection-induced brain overgrowth. In addition, an imbalance in redox regulation involving NAPDH oxidase activation and increases in reactive oxygen species in the NSCs of the postnatal subventricular zone is implicated in the mechanisms underlying brain overgrowth and ASD-like behaviour phenotypes [63]. In the present study, we observed upregulation of oligodendrocyte differentiation marker gene *Olig2* when LPS was administered to pregnant mice on GD 9 or to cells during the ESC–NSC transition in vitro, indicating aberrant oligodendrocyte differentiation from NSCs. Moreover, the prefrontal cortex tissues showed a high lipid metabolism gene score (Fig. 2B), which was calculated using NanoString based on the mRNA expression of lipid metabolism genes, particularly *ApoE* (Fig. 2C). ApoE can induce NSCs and neural progenitor cells to differentiate into additional oligodendrocytes [72] and facilitate myelination and oligodendrogenesis after stroke [73]. Consistent with other studies [72, 73], we observed a higher abundance of CNPase^+^ oligodendrocytes (Fig. 3C-i) or O4^+^ oligodendrocytes (Fig. 3E) in the prefrontal cortex and striatum regions of the male offspring of the pregnant mice exposed to LPS on GD 9. Elevated neuropsychiatric symptoms has been associated with poor visual short-term and long-term memory in ApoE^+^ humans [74].

Similar to the findings regarding the involvement of *ApoE* in oligogenesis and myelination in vivo (Fig. 2), our in vitro findings demonstrated that *ApoB* was upregulated in the differentiated neural cells of LPS-treated NSCs (Table 1). Astrocytes and microglia express *ApoE* in the brain, and LPS stimulation increases microglia-secreted ApoE levels [51]. However, research has revealed that *ApoB*-derived peptides can exert antimicrobial activities, including antibiofilm, wound-healing and immunomodulatory activities, and even function synergistically with some antibiotics [75]. In contrast, ApoB levels are significantly higher in patients with bipolar disorder compared with those with unipolar disorder, making ApoB a potential biomarker for distinguishing between the two [76]. In addition, ApoB content in cerebral spinal fluid is strongly associated with early tau dysregulation in the development of visuospatial cognitive disorders [77]. Furthermore, LPS may disrupt the endothelial blood–brain barrier along with oxidised cholesterol levels and intensify ApoB’s immunogenicity. This may explain the mechanism underlying neuro-inflammation resulting from chronic infections [78].

Our results suggest that early prenatal challenges with LPS differentially induce anxiety-like behaviour in male offspring. This anxiety-like behaviour may result from LPS-induced aberrant astrocyte and oligodendrocyte differentiation in the brain, potentially through immune responses mediated by the *ApoB*/*E* receptor signalling axis. Moreover, the timing of prenatal infection may predict the neural disorder type and severity in adulthood. Despite our interesting findings mentioned above, there are two main limitations of our study: (i) the sample sizes of some experiments, such as behaviour tests and neurosphere formation assay, can be increased to improve statistical significance of our findings; and (ii) we only assessed the difference in open field tests and used it as an indication of anxiety-like behaviour. Nonetheless, our data suggest that prenatal exposure to LPS has differential effects on the FVB mouse strain, providing a valuable comparison with the studies in the literature [61–63]. Taken together, our findings may serve as a basis for understanding abnormal glial differentiation in response to prenatal LPS exposure. This understanding could potentially facilitate the development of prevention and therapeutic strategies to mitigate the adverse neurological effects of prenatal infection.

### Supplementary Information

**Additional file 1:** Approved Animal Protocol MMH-A-S-106-52.

## Data Availability

The datasets used and/or analysed during the current study are available from the corresponding author on reasonable request.

## Abbreviations

ASD: Autism spectrum disorders
ESCs: Embryonic stem cell
EZM: Elevated zero maze
EGF: Epidermal growth factor
FGF2: Fibroblast growth factor 2
FITC: Fluorescein isothiocyanate
GD: Gestation days
HRP: Horseradish peroxidase
IL: Interleukin
i.p.: Intraperitoneally
IFN: Interferon
MTT: 3-[4,5-Dimethylthiazol-2-yl]-2,5 diphenyltetrazolium bromide
NSCs: Neural stem cells
PBS: Phosphate-buffered saline
TNFα: Tumour necrosis factor α
