# Supplementary material for: Prenatal lipopolysaccharide exposure induces anxiety-like behaviour in male mouse offspring and aberrant glial differentiation of embryonic neural stem cells
Source: Cell Mol Biol Lett. 2023 Aug 17;28:67. doi: 10.1186/s11658-023-00480-7 (PMC10436442; doi:10.1186/s11658-023-00480-7)
Supplement: Supplementary file 1 — Additional file 1: Approved Animal Protocol MMH-A-S-106-52. [file 11658_2023_480_MOESM1_ESM.docx]

**Additional file 1**

**Table S1. Primer sets for real-time PCR.**

| Primer | Sequence | Product Size (bp) |
| --- | --- | --- |
| Oct4-F | **5’-TCAGGTTGGACTGGGCCTAGT-3’** | 99 |
| Oct4-R | **5’-GGAGGTTCCCTCTGAGTTGCTT-3’** |  |
| Sox2-F | **5’-AGATGCACAATCCGGAGATCAG-3’** | 62 |
| Sox2-R | **5’-GCTTCTGGTCTCGGACAAA-3’** |  |
| Sox1-F | **5’-TGAAGGAACACCCGGATTACA-3’** | 79 |
| Sox1-R | **5’-GCCAGCGAGTACTTGTCCTTCTT-3’** |  |
| Nestin-F | **5’-AGCCTGGAAAGGAACCAAAAG-3’** | 74 |
| Nestin-R | **5’-CTCACTGCTAGTCTTGGGTAATTCAG-3’** |  |
| Tuj1-F | **5’-ATGAGGGAGATCGTGCACATC-3’** | 65 |
| Tuj1-R | **5’-TCCCAGAACTTGGCCCCTAT -3’** |  |
| Map2-F | **5’-TCCTCCAAAGTCCCCAGCTA-3’** | 65 |
| Map2-R | **5’-TCAGGTCCGGCAGTGGTT-3’** |  |
| Gfap-F | **5’-CGGAGAcGCATCACCTCTG-3’** | 126 |
| Gfap-R | **5’-AGGGAGTGGAGGAGTCATTCG-3** |  |
| O4-F | **5’-CTCGCCCTGTACCGAAGGA-3’** | 152 |
| O4-R | **5’-CACTTGCCGTACTGACGGATG-3’** |  |
| Oligo2-F | **5’-TCCCAGAACCCGATGATCTT-3’** | 88 |
| Oligo2-R | **5’-CGTGGACGAGGACACAGTC-3** |  |
| TLR2-F | **5’-ATTTCCACGGACTGTGGTACCT-3** | 70 |
| TLR2-R | **5’-GCTTTCTTGGGCTTCCTCTTG-3’** |  |
| TLR4-F | **5’-TGACAGGAAACCCTATCCAGAGTT-3’** | 81 |
| TLR4-R | **5’-TCTCCACAGCCACCAGATTCT-3’** |  |
| 18s-F | **5’-CGAGCCGCCTGGATACC-3’** | 76 |
| 18s-R | **5’-CCTCAGTTCCGAAAACCAACAA-3’** |  |

**Fig. S1**

**(A)**


**(B)**

**(C)**

**Fig. S1. Effects of prenatal exposure of LPS on the body weight of male and female offspring. (A) Distribution of experimental mice by sex and litter size. Blue bar: male, red bar: female, black bar: total. Prenatal LPS exposure in pregnant mice did not affect the body weights of male (B) and female (C) offspring.**

**Fig. S2**


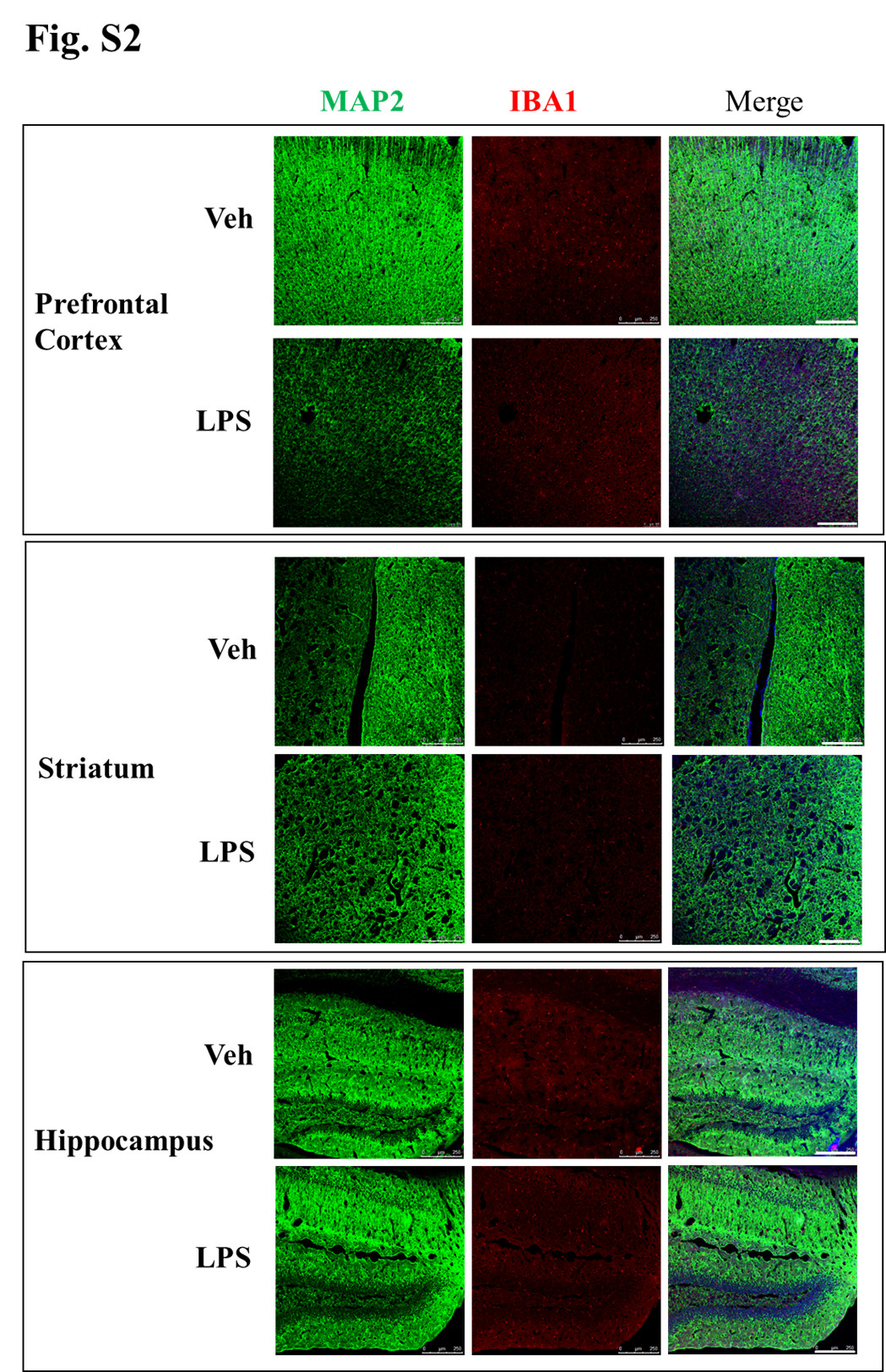


**Fig. S2. Immunohistochemistry for microglia in different brain regions of male offspring with prenatal LPS exposure.** Representative images from the immunohistochemical double staining experiments revealing MAP2^+^ and IBA1^+^ cells in prefrontal cortex, striatum, and hippocampus tissues. Scale bar = 250 µm.

**Fig. S3**


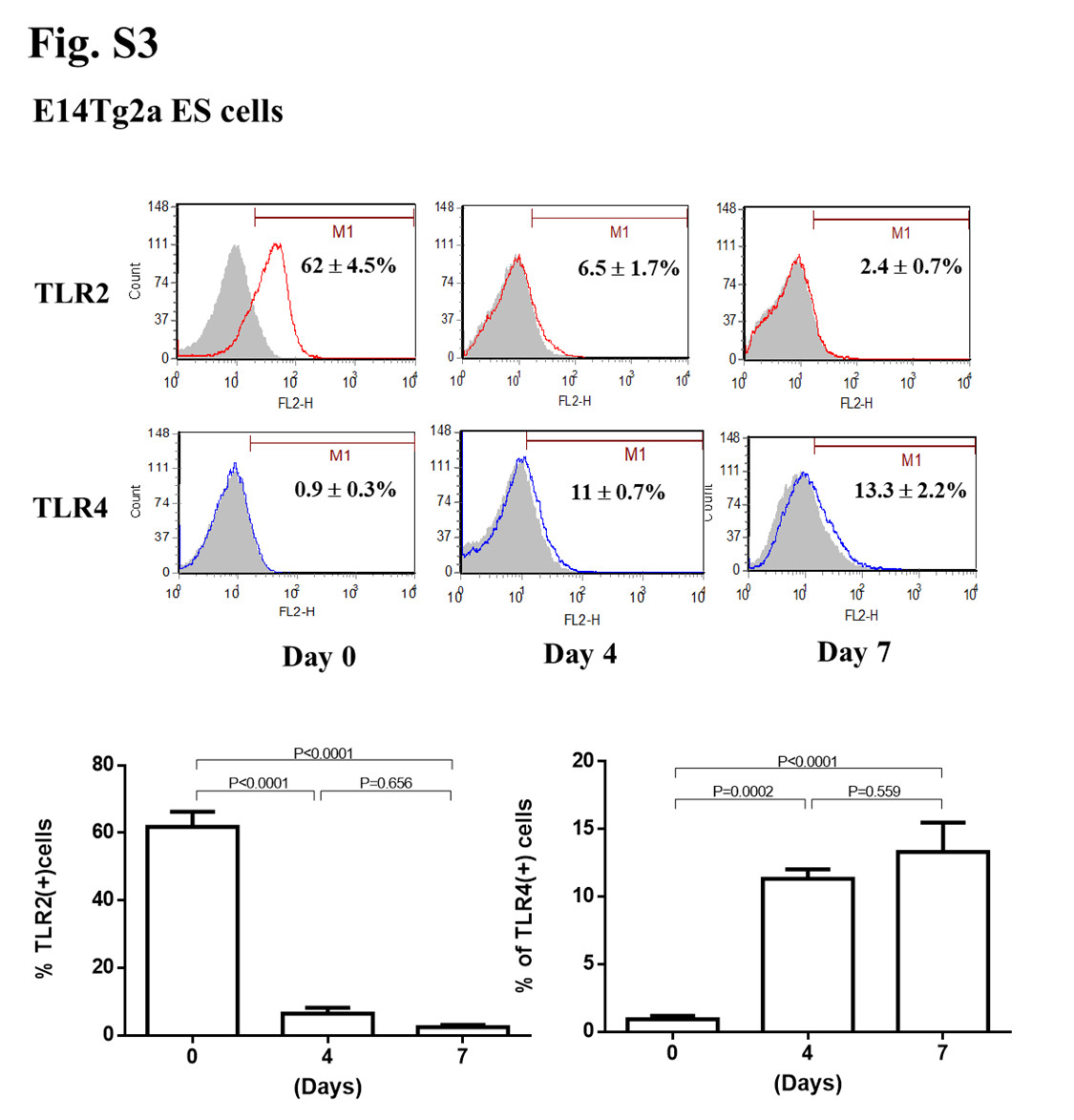


**Fig. S3.** Toll-like receptor (TLR) 2 and TLR4 expression in E14Tg2a cells during ESC-to-NSC transition and NSC-to-neural differentiation transition. (A) Flow cytometry and (B) quantification of TLR2^+^ and TLR4^+^ ESCs and ESC-derived NSCs. Data are shown as means ± SEM, *n* ≥ 6.

**Fig. S4**


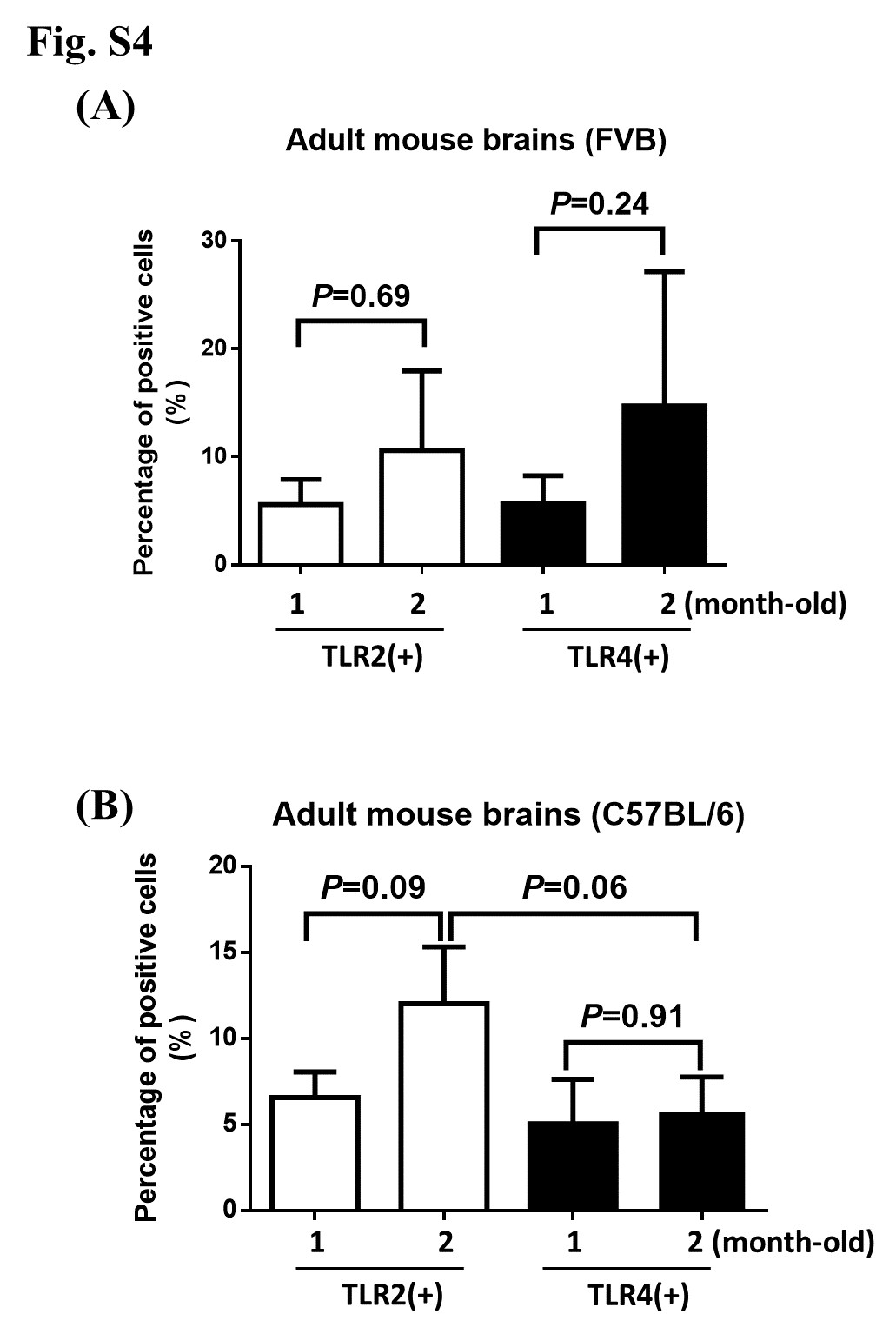


**Fig. S4.** Quantification of toll-like receptor (TLR) 2-positive and TLR 4-positive cells in adult 1-month-old and 2-month-old (A) FVB and (B) C57BL/6 mouse brains. Data are presented as means ± SEM (*n* ≥ 3).
